# Retraction Notice to: Modulation of Platelet Functions Assessment during Menstruation and Ovulatory Phases

**DOI:** 10.25122/jml-2026-1011

**Published:** 2026-06

**Authors:** Faisal Alzahrani, Fathelrahman Hassan

**Affiliations:** 1 Department of Clinical Laboratory Science, Imam Abdulrahman Bin Faisal University, College of Applied Medical Science, Dammam, Saudi Arabia

## Abstract

Article J Med Life 2019; Jul-Sep;12(3):296-300; PubMed ID: 31666834 has been retracted

## RETRACTION

The Editors of Journal of Medicine and Life are retracting the article “Modulation of Platelet Functions Assessment during Menstruation and Ovulatory Phases” by Faisal Alzahrani and Fathelrahman Hassan (Vol. 12, Issue 3, 2019, pp. 296–300; DOI: 10.25122/jml-2019-0005).

The retraction follows Decision No. 07/24 of the Copyright Protection Lawsuits Committee of the Saudi Authority for Intellectual Property, which found that the article reproduced protected material without the authorization or attribution of the rights holder, and ordered its removal.

The full text has been withdrawn from public access and replaced with this notice. The bibliographic metadata and DOI are retained to preserve the scholarly record.
